# Absorbable calcium and phosphorus bioactive membranes promote bone marrow mesenchymal stem cells osteogenic differentiation for bone regeneration

**DOI:** 10.1515/biol-2022-0854

**Published:** 2024-04-16

**Authors:** Lei Huang, Zhuorun Song, Jiayi Wang, Mengxuan Bian, Jiapeng Zou, Yanpei Zou, Jun Ge, Shunyi Lu

**Affiliations:** Department of Orthopedic Surgery, The First Affiliated Hospital of Soochow University, Suzhou, Jiangsu, 215006, China; Department of Orthopedic Surgery, Zhongshan Hospital, Fudan University, Shanghai, 200032, China; Shanghai Jiao Tong University Affiliated Sixth People’s Hospital, Shanghai 200233, China

**Keywords:** biodegradable PLGA nanofiber, β-TCP, osteogenesis, angiogenesis

## Abstract

Large segmental bone defects are commonly operated with autologous bone grafting, which has limited bone sources and poses additional surgical risks. In this study, we fabricated poly(lactide-*co*-glycolic acid) (PLGA)/β-tricalcium phosphate (β-TCP) composite membranes by electrostatic spinning and further promoted osteogenesis by regulating the release of β-TCP in the hope of replacing autologous bone grafts in the clinical practice. The addition of β-TCP improved the mechanical strength of PLGA by 2.55 times. Moreover, β-TCP could accelerate the degradation of PLGA and neutralize the negative effects of acidification of the microenvironment caused by PLGA degradation. *In vitro* experiments revealed that PLGA/TCP10 membranes are biocompatible and the released β-TCP can modulate the activity of osteoblasts by enhancing the calcium ions concentration in the damaged area and regulating the pH of the local microenvironment. Simultaneously, an increase in β-TCP can moderate the lactate content of the local microenvironment, synergistically enhancing osteogenesis by promoting the tube-forming effect of human umbilical vein endothelial cells. Therefore, it is potential to utilize PLGA/TCP bioactive membranes to modulate the microenvironment at the site of bone defects to promote bone regeneration.

## 1 Introduction

Although natural bone tissue has the ability to self-repair small defects, severe bone defects due to trauma, tumor resection, and congenital malformations mostly require clinical intervention [1,2]. Autologous bone grafting is a common surgical procedure used in practice, but has obvious limitations due to deficiencies in donor resources and the risk of secondary injuries [3,4]. Previous studies have explored various methods to prepare bone repair membranes, with reports showing fibrous membranes made using electrostatic spinning being popular in the biomedical field due to their high porosity, drug loading properties, and biomimetic topology [5,6]. In the recent decades, bone repair membranes are commonly prepared by 3D printing, Hot-Press compression molding, and electrostatic spinning [7–9]. Fibrous membranes prepared though electrostatic spinning was reported to have high porosity, drug-loading properties and biomimetic topology, and is widely studied in the biomedical field [10,11]. Bioactive bone membranes are supposed to have several criteria including biocompatibility, osteoinductive activity, and space maintenance capacity, further promoting bone regeneration and rebuilding bone microenvironment [12,13]. Additionally, bioactivity can be considered a desirable feature of bone regeneration membrane in order to initiate and enhance bone formation [14]. Hence, artificial bone graft substitute is considered as an alternative to autologous/allograft. It is of great significance to develop a biocompatible and osteoinductive activity bone membrane for the clinical treatment of bone defects.

Poly(lactide-*co*-glycolic acid) (PLGA) has been approved by Food and Drug Administration (FDA) for clinical therapeutic use as a copolymer with controlled biodegradability and good biocompatibility [15]. It has been widely used for drug delivery due to the slow-releasing properties of the structure [16]. Jadidi et al. [17] showed encapsulating vancomycin into a PLGA coating for slow release significantly improved the cellular activity of the scaffold. Meanwhile, the lactic acid released from PLGA degradation can be metabolized by the body and promotes angiogenesis [18]. Study has indicated that PLGA microspheres are beneficial to implant *in situ* pore formation and bone ingrowth in bone defect areas [15]. β-tricalcium phosphate (β-TCP) is an absorbable calcium-phosphate salt that is susceptible to new bone replacement and is widely applied in bone repair because of its good osteoconductive capacity. Due to its similarity to bone minerals in terms of crystallinity and chemical composition, β-TCP has been favored by researchers for its excellent biocompatibility and biodegradability [19]. Incidentally, studies also demonstrated that PLGA can combine well with β-TCP to prepare various complexes to facilitate bone regeneration [20,21].

In this study, we intended to prepare PLGA/β-TCP composite membranes by electrostatic spinning to control the release of β-TCP. The mechanical and thermal properties as well as the *in vitro* osteogenic and angiogenic abilities of the composite membranes were then evaluated. The pore formation with the addition of PLGA in β-TCP promoted the growth of new bone and vascularization property. Our results reveal that PLGA/β-TCP composite membranes can be the next generation of bone regenerative material.

## 2 Materials and methods

### 2.1 Preparation of PLGA/β-TCP bioactive membrane

600 mg PLGA (lactic acid, LA:glycolic acid, GA = 70:30, *M*_w_ = 10^5^, DaiGang, China) was added to 4 mL of tetrahydrofuran/N,N-dimethylformamide (v/v = 3:1) mixed solution and stirred magnetically at room temperature for 24 h. Then, 6, 30, and 60 mg β-TCP (Sigma, USA) were added to the PLGA solution and stirred for another 3 h before sonication for 1 h. The solution was sonicated at an operating voltage of 22 kV, operating distance of 15 cm, and a feed rate of 1 mL/h. Samples were named PLGA, PLGA/TCP1, PLGA/TCP5, PLGA/TCP10 according to different β-TCP concentrations.

### 2.2 Membrane characterization

#### 2.2.1 Surface morphology

Scanning electron microscopy (SEM, S-4800, Hitachi, Japan) was used to analyze the surface morphological characteristics of the membrane specimens. Elemental analysis was conducted by energy dispersive spectroscopy, and mapping of both calcium and phosphorus. β-TCP particles were analyzed by transmission electron microscopy (TEM, Hitachi, H-800, Japan).

#### 2.2.2 Chemical phase composition

The bioactive membrane specimens were investigated by X-ray diffraction (XRD, Rigaku D/Max2550, Cu Karadiation, Japan).

#### 2.2.3 Differential scanning calorimetry analysis

The thermal properties of the samples were analyzed by differential scanning calorimetry (DSC2910, USA). The test conditions were: 3–5 mg samples were heated from 25 to 200°C at 10°C/min to eliminate thermal history, held at 200°C for 2 min, then cooled to 0°C at 10°C/min held at 0°C for 2 min, then heated to 220°C at 10°C/min.

#### 2.2.4 Thermogravimetric analysis

The thermal stability of the specimens was tested by a thermogravimetric analyzer (TG209F1-GC 7820A, China), where 3–5 mg of specimens would be heated from 30 to 600°C in nitrogen at a rate of 10°C/min.

#### 2.2.5 Degradation characteristics

The degradation characteristics of the samples were performed in phosphate buffered saline (PBS). For each group of three parallel samples (30 mm × 10 mm × 1 mm), they were submersed in 10 mL of PBS and placed in a shaker (37°C, 80 rpm). The pH of the soaking solution was measured with a pH meter (Mettler Toledo, China) for the corresponding days and change in mass was measured.

#### 2.2.6 Ion release

The pH changes and ion release of the samples were performed in PBS. For each group of three parallel samples (30 mm × 10 mm × 1 mm), they were soaked in 10 mL of PBS and placed in a shaker (37°C, 80 rpm). The collected soaking solution was measured with an inductively coupled plasma atomic emission spectrometer (ICP-AES, PerkinElmer Optima 2000, USA) for Ca^2+^ concentration.

#### 2.2.7 Mechanical properties

The mechanical properties of the composite film were determined by a universal mechanical testing machine (SANS CMT 2503, USA), which was tested in room temperature at a stretching speed of 10 mm/min.

### 2.3 *In vitro* osteogenesis assay

#### 2.3.1 Cell culture

Protocol of animal experimentation in the study was approved by the Animal Care and Use Committee of Suzhou University (Suzhou, China). Four-week-old Sprague-Dawley rats (SD rats) were purchased from Shanghai JASJ Laboratory Animal Co. Primary bone marrow mesenchymal stem cells (BMSCs) were derived from the femur and tibia of the SD rats in accordance with the published methodology [15]. BMSCs were cultivated in α-MEM medium (Gibco, USA) containing 10% fetal bovine serum, (BI, USA) and 1% penicillin and streptomycin (Sigma, USA). Cells of the second to fourth generation were used for subsequent experiments. All material samples were sterilized by ethylene oxide and isolated samples (10 mm × 3 mm) were immersed in α-MEM at 37°C for 24 h to retrieve the material extracts following ISO 10993.


**Ethical approval:** The research related to animal use has been complied with all the relevant national regulations and institutional policies for the care and use of animals, and has been approved by the Animal Care and Use Committee of Suzhou University.

#### 2.3.2 Cell proliferation and adhesion

To perform live/dead staining assays, BMSCs were processed with material extract for 1/3/5/7 days and then exchanged for a Calcein-AM/PI double staining kit (Beyotime, China) for 30 min according to the instruction manual. Observation and photography were carried out with an inverted fluorescent microscope (Olympus, IX71, Japan).

Cell counting kit-8 (CCK-8) assay of BMSCs was conducted with the density of 5 × 10^4^ cells/well seeded in a 24-well culture plate. After 1, 3, and 5 days of incubation with material extract, the medium was substituted with α-MEM medium containing 10% CCK-8 solution (Beyotime, China). The plates were then cultured at 37°C and 5% CO_2_ for 30 min, and the absorbance on the plates was recorded at 450 nm using a microplate reader (Epoch, BioTek Instruments, Inc.).

#### 2.3.3 Osteogenic differentiation

To study osteogenic differentiation, osteogenic induction medium (OIM) was made of α-MEM containing 0.05 mM vitamin C (Sigma, USA), 10 mM β-glycerophosphate (Sigma, USA), and 10 nM dexamethasone (Sigma, USA). BMSCs were prepared in 12-well plates along with extracts in a cell culture incubator at 1 × 10^5^ cells/well. The medium was substituted with OIM upon reaching cell density confluency of 70–80%. BMSCs culture were exchanged for OIM every 3 days to evaluate osteogenic differentiation with alkaline phosphatase (ALP) staining, ALP activity was recorded after 14 days of culture and Alizarin Red S (ARS) staining after 21 days.

A BCIP/NBT solution (Beyotime, China) was utilized to determine ALP expression. After 14 days of incubation, BMSCs were washed three times with PBS and then processed with 10% neutral formalin for 15 min. Samples were stained according to the protocol and washed with PBS before observation with an optical microscope (Olympus, IX71, Japan). After 14 days of incubation, ALP activity was examined using an ALP assay kit (Beyotime, China). BMSCs were lysed with RIPA lysis buffer (Beyotime, China) and then centrifuged for 20 min at 3,000 rpm to detect ALP activity. Total cell protein was quantified in parallel with the BCA protein assay kit (Beyotime, China). Activity of ALP was identified after absorbance measurements at 405 nm on a microplate reader (Epoch, BioTek Instruments, Inc.)

ARS staining assay was performed to assess mineralization (Beyotime, China). Following 21 days of culture, calcium deposition was assessed with 0.5% Alizarin Red workup and observed by optical microscopy according to the instruction manual (Olympus, IX71, Japan).

### 2.4 *In vitro* angiogenesis assay

#### 2.4.1 Cell culture

Human umbilical vein endothelial cells (HUVECs) were acquired from the Shanghai Institutes for Biological Sciences cell bank and were utilized to estimate the influence of β-TCP on angiogenesis *in vitro*.

#### 2.4.2 Tube formation assay

Twenty-four-well plates were pre-treated initially with polymerized Matrigel (BD Biosciences, San Jose, CA). Cells were then seeded onto the Matrigel surface at a density of 1 × 10^5^ cells/well and incubated in the extract for 12 h. Microscopic images were taken at random and ImageJ with the Angiogenesis Analyzer plug-in (NIH, Bethesda, MD) was used to quantify the tubular network (number of primary nodes and total segment length).

#### 2.4.3 Transwell assay

The invasion capacity of HUVECs was assayed by the transwell assay. HUVECs were placed on the top layer of a transwell cell culture chamber (Costar, Cambridge, MA, USA). After the transwell chamber was capped with 50 μL of Matrigel (BD, Biosciences, USA), the membrane extract was placed underneath the cell permeable membrane for 12 h of incubation. The upper chamber was then washed twice with HUVECs to exclude non-invasive cells. HUVECs that had migrated through the membrane were fixed with 4% paraformaldehyde (Beyotime, China) for 15 min and then colored with 0.4% crystal violet stain (Amresco, USA) for 20 min. Several microscopic areas were randomly selected for analysis.

#### 2.4.4 Angiogenesis-related gene levels

After 3 days in culture, HUVECs were harvested and reverse transcribed to cDNA and RT-PCR was performed to check the gene expression levels of Willebrand Factor (vWF), vascular endothelial growth factor (VEGF), CD31, and β-actin as a control reference. Sequence information used in the study is presented in Table 1.

**Table 1:** Primer sequences of each angiogenesis-related gene

Gene	Direction	Sequence (5′–3′)
VEGF	Forward	TTCAAGCCATCCTGTGTGCC
	Reverse	CACCAACGTACACGCTCCAG
vWF	Forward	CCGATGCAGCCTTTTCGGA
	Reverse	TCCCCAAGATACACGGAGAGG
CD31	Forward	AACAGTGTTGACATGAAGAGCC
	Reverse	TGTAAAACAGCACGTCATCCTT
β-actin	Forward	ATGTTCCAGTATGACTCCACTCAC
	Reverse	GAAGACACCAGTAGACTCCACGAC

### 2.5 Statistical analysis

At least three independent replicates were used for each experiment and continuous variables were represented as mean value ± standard deviation. One-way analyses of variance (ANOVA) were used to compare different treatment interventions in SPSS 20.0. The level of significant difference was set at p* < 0.05 and p** < 0.01.

## 3 Results

### 3.1 Chemical phase and thermal properties of the active membranes

**Figure 1:**
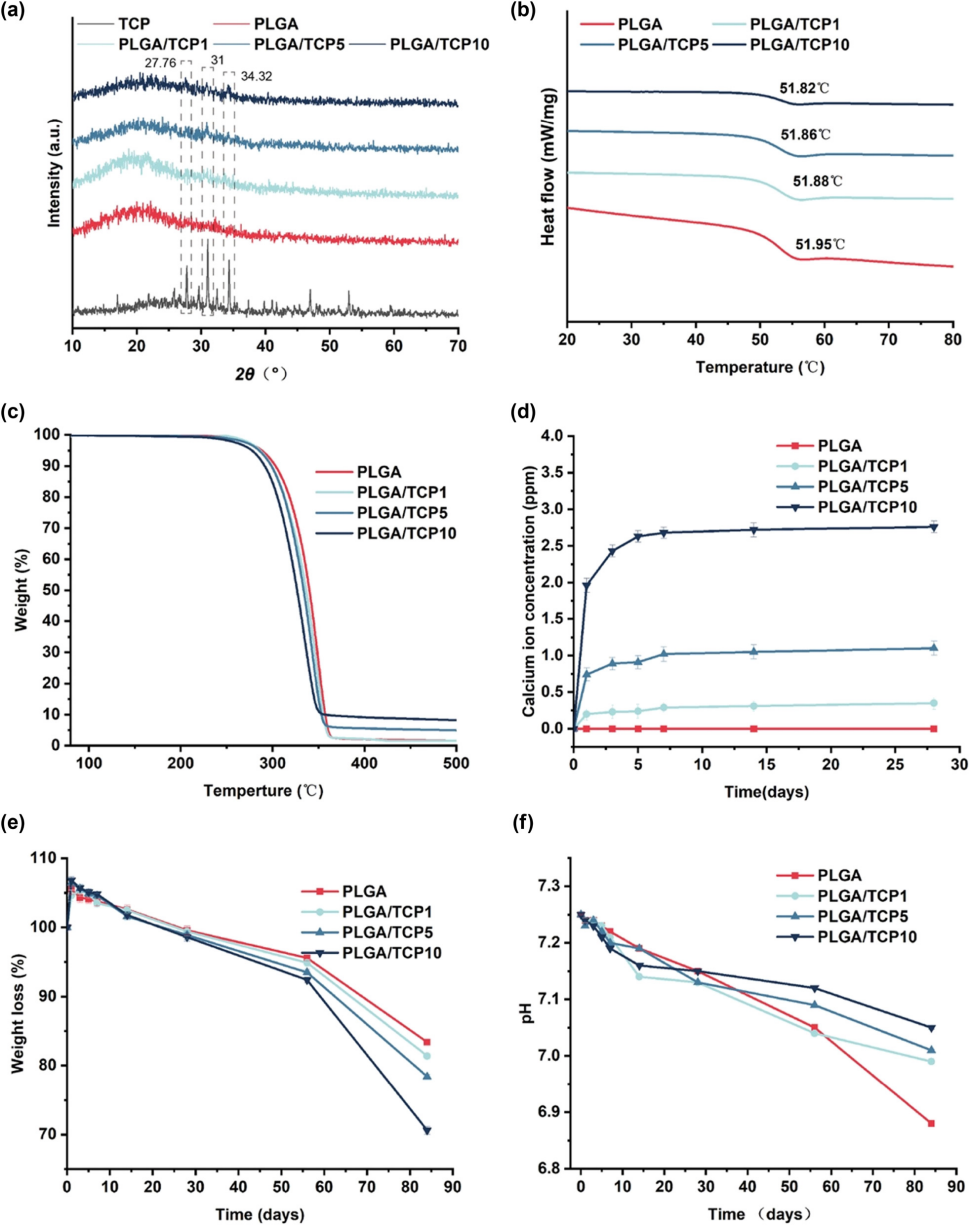
Chemical phase and thermal properties of the active membranes. (a) XRD of the membrane. (b) DSC. (c) TGA. (d) Ca^2+^ release concentration. (e) Weight loss and (f) pH vibration of PBS after samples soaking. Data are presented as the mean value ± SD; *n* = 3.

As shown in Figure 1a, all groups of samples exhibited a broad peak at 2*θ* = 22°. PLGA is an amorphous polymer and the intensity of this peak with concentration of β-TCP content increases, indicating that β-TCP was well dispersed within the PLGA films in this concentration range. Loading of β-TCP in the PLGA membrane is shown in Figure 1b and c. The incorporation of β-TCP caused a decrease in the thermal decomposition temperature and glass transition temperature of PLGA, but the effect on thermal performance was slight. The decrease in thermal stability may be due to the agglomeration of β-TCP particles. The regularity of ion release also remained largely constant in the composite membrane. Concentration of calcium ions released best was in first 24 h and reached a stable value after 2 weeks, depending on the amount of β-TCP (Figure 1d). The addition of β-TCP not only accelerated the degradation of PLGA, but also neutralized the negative side effects of acidification of the microenvironment caused by PLGA degradation (Figure 1e and f).

### 3.2 Mechanical properties of bioactive membrane

**Figure 2:**
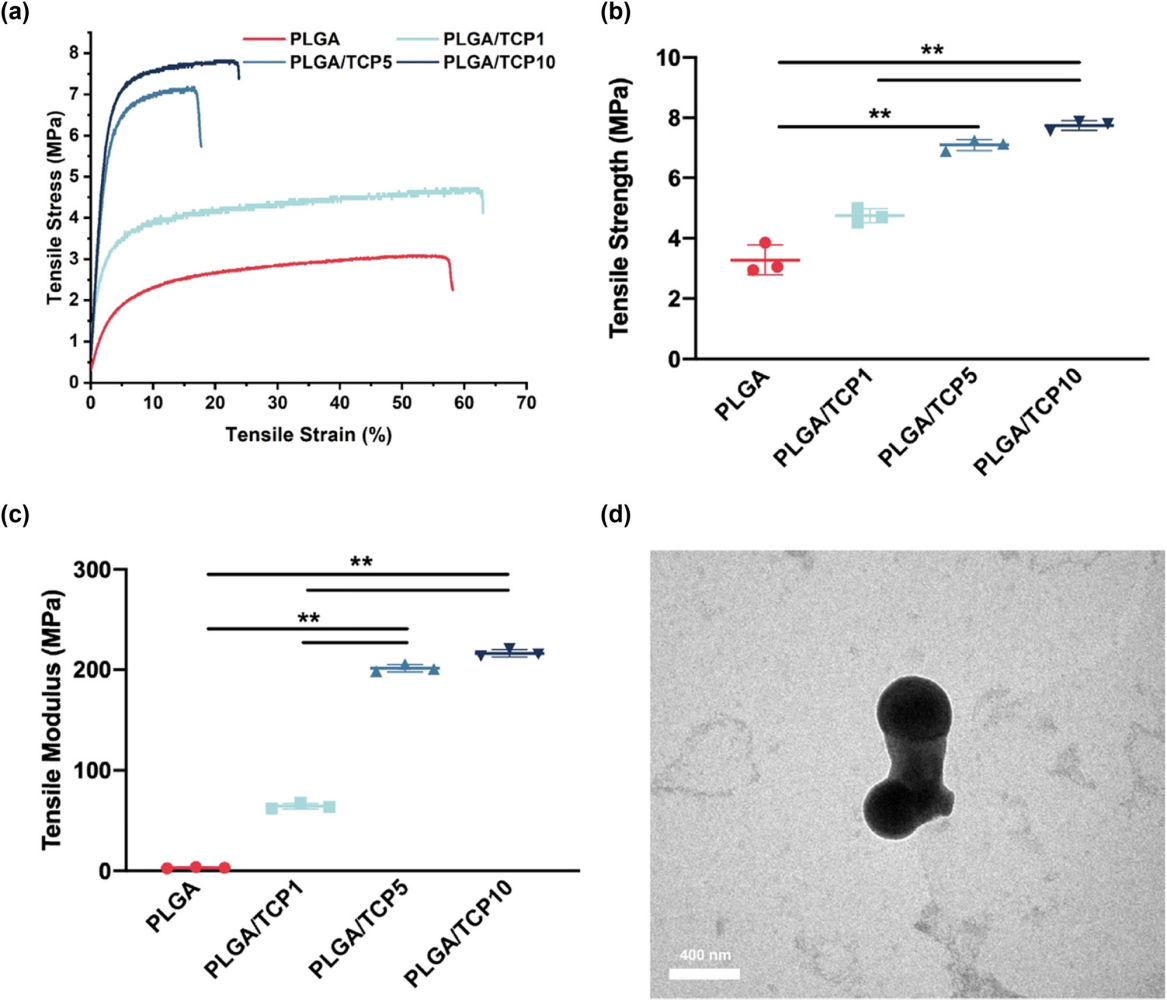
The mechanical properties of PLGA/β-TCP bioactive membrane. (a) Tensile stress–tensile strain of PLGA/β-TCP bioactive membrane, (b) tensile stress, and (c) tensile modulus. (d) Microscopic morphology of β-TCP. Data are presented as the mean value ± SD; *n* = 3; *p < 0.05 and **p < 0.01.

Mechanical properties of the electrospinning membrane increased continuously with the increase in β-TCP content, from the initial 3.05 ± 0.10 MPa (PLGA) to 7.79 ± 0.135 MPa (PLGA/TCP10). The tensile strength of PLGA/TCP5 film increased by 134% compared to the pure PLGA film, but the β-TCP content increased from 5 to 10 wt%, mechanical properties only improved by 9% (Figure 2a and b). The tensile modulus displayed the same trend in Figure 2c.

### 3.3 Microscopic morphology and elemental distribution of the membranes

**Figure 3:**
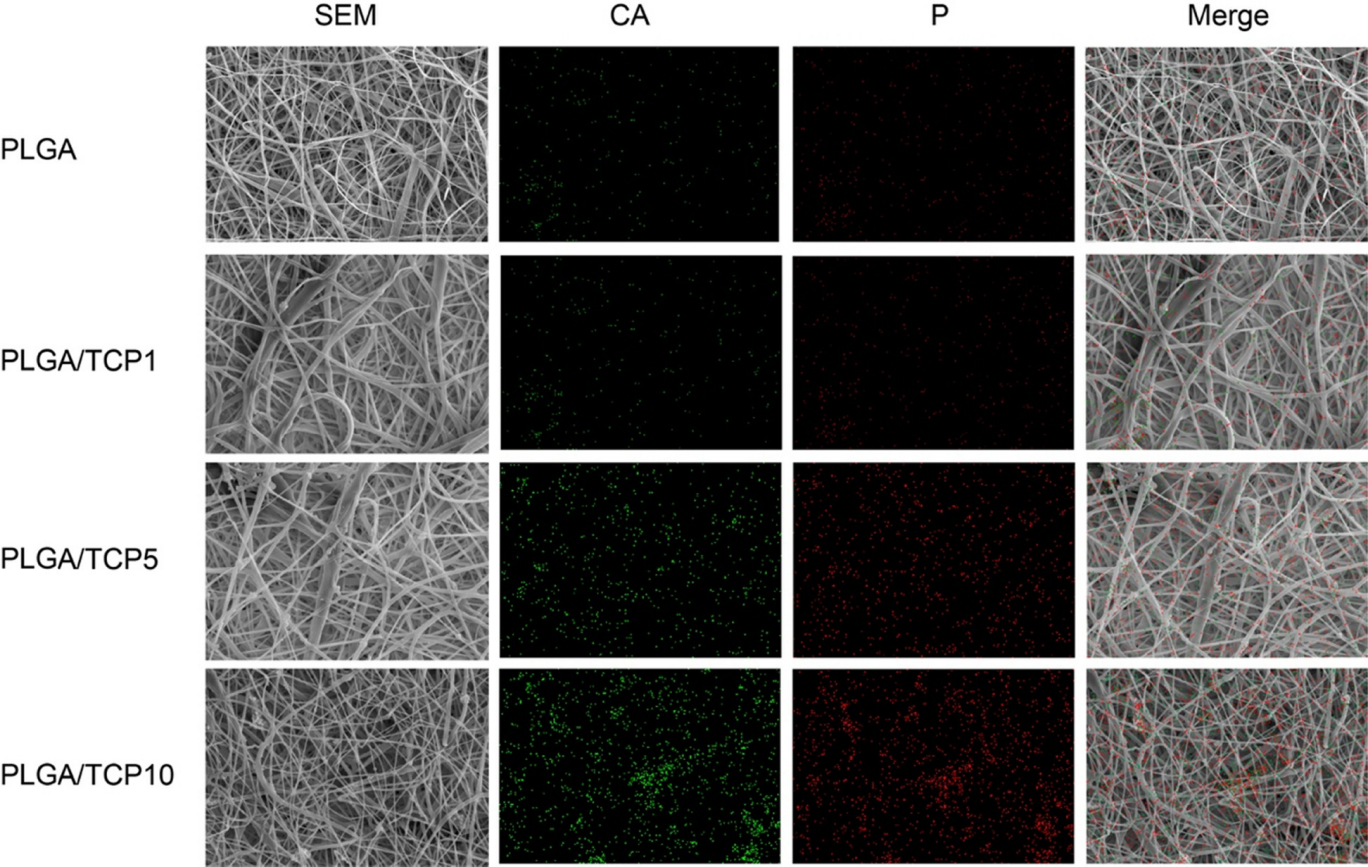
Microscopic morphology and elemental distribution of the membranes. First column: SEM of the membrane; second column: elemental distribution of calcium; third column: elemental distribution of phosphorus; fourth column: elemental distribution on the membrane.

The microscopic morphology of the composite film can be clearly observed from Figure 3. Meanwhile, the roughly 400 nm of β-TCP was observed by the TEM (Figure 2d). The addition of β-TCP did not have much effect on the diameter of the PLGA fibers, which remained in the range of 1–20 μm. As shown in the elemental distribution diagram, β-TCP is well dispersed in the PLGA fibers. The dense membrane structure has the potential to act as a physical barrier at the wound site [22].

### 3.4 Effect of PLGA/TCP on proliferation and adhesion of BMSCs

**Figure 4:**
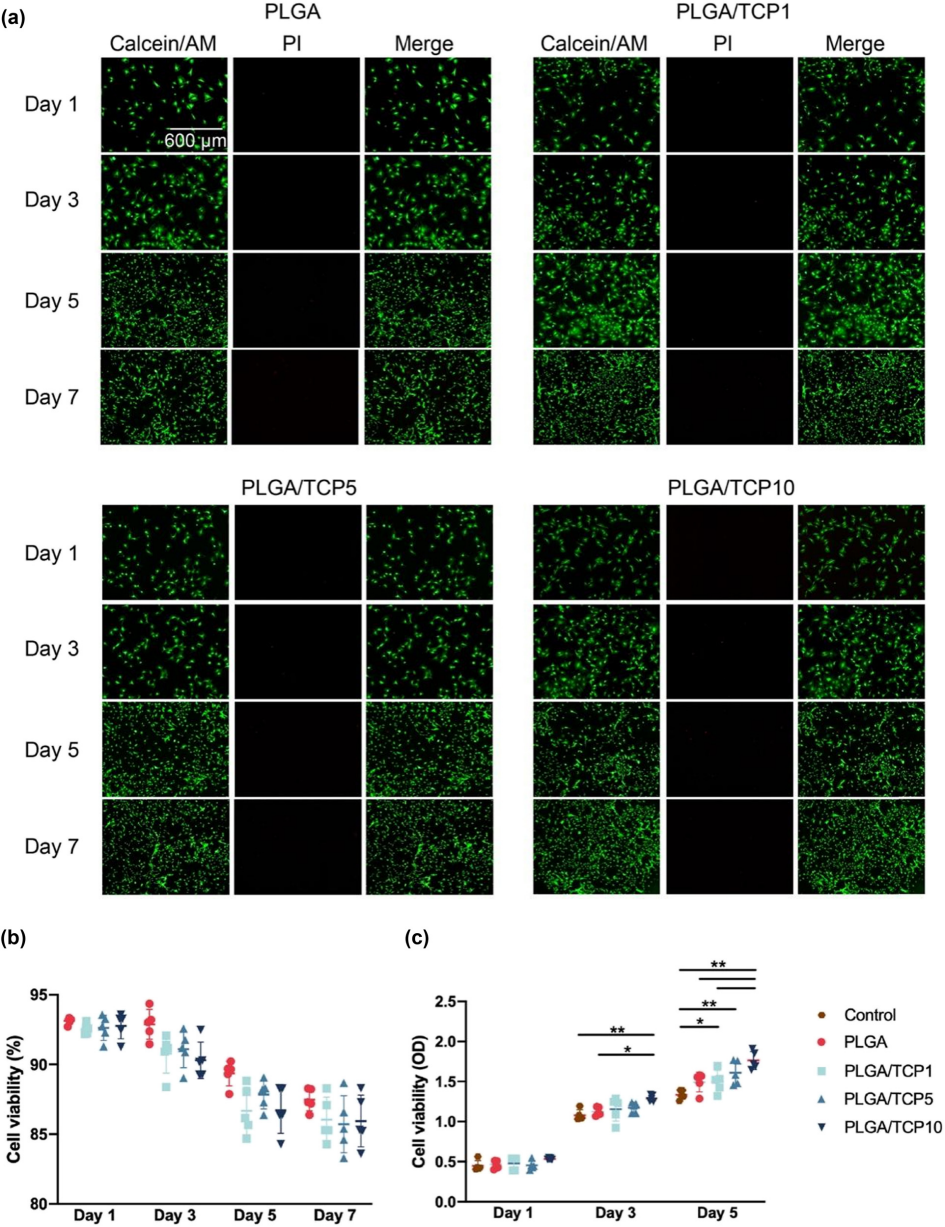
Proliferation and cell adhesion of BMSCs treated with extracts. (a) Live/dead staining results of BMSCs. (b) Cell viability according to the live/dead assays. (c) CCK-8 analysis of BMSCs. Data are presented as mean value ± SD; *n* = 3; *p < 0.05 and **p < 0.01.

Cell viability is a primary parameter for analyzing the biocompatibility of a bio-scaffold as it directly affects both cell proliferation and differentiation directly. We cultivated BMSCs with PLGA/TCP extracts. Live/dead cell staining revealed that the scaffold did not hinder cell proliferation and almost no dead cells were detected during the observation (Figure 4a and b). Cell viability was measured in parallel using the CCK-8 assay to facilitate assessment. As shown in Figure 4c, the OD values increased with incubation time, but there was no statistical difference between the three groups, demonstrating that BMSCs proliferated well in the infiltrates of the material in each group. Additionally, we co-cultured cells with the surface of the material and detected the cytotoxic effect of materials on both BMSCs and HUVECs by using live/dead staining and CCK-8 assays after 1, 4, and 7 days. The results also showed that the bioactive membranes had no cytotoxic effect after co-culturing cells on the surface of the materials (Figures S2 and S3). In summary, PLGA/TCP was non-cytotoxic and promoted cell adhesion and proliferation.

### 3.5 Effect of PLGA/TCP on osteogenic differentiation capacity of BMSCs

**Figure 5:**
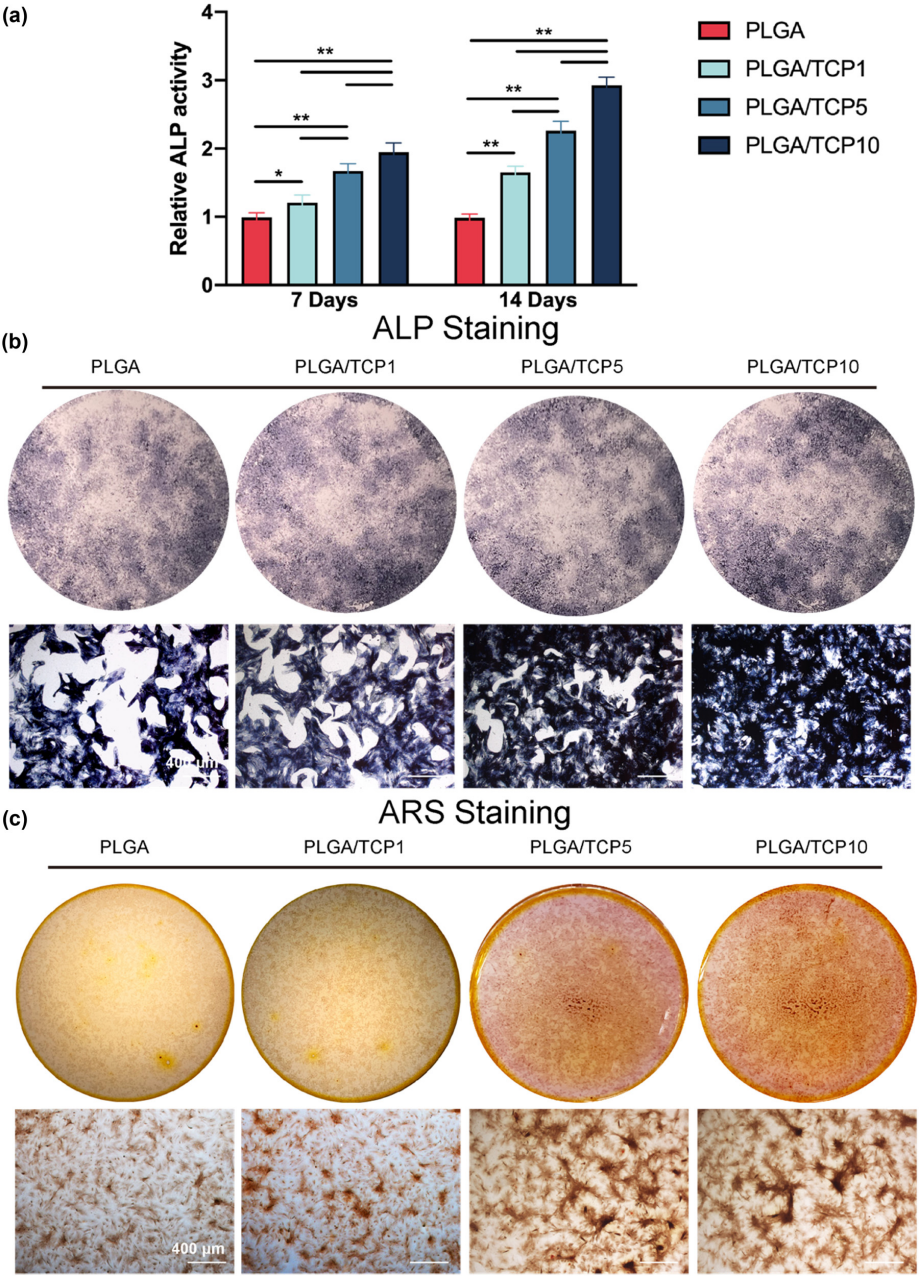
Osteogenic effect on BMSCs treated with extracts. (a) Quantification of ALP activity of BMSCs for 7 and 14 days. (b) ALP staining of BMSCs for 14 days. (c) ARS staining of BMSCs for 21 days. Data are presented as mean value ± SD; *n* = 3; *p < 0.05 and **p < 0.01.

To verify the subsequent effect of PLGA/TCP on osteogenesis in the local microenvironment, we combined the material extract with OIM to culture BMSCs. Figure 5a and b illustrate the outcome of ALP staining and ALP activity of BMSCs after 14 days of culture. ALP staining revealed an increased amount of ALP-positive cells in the PLGA/TCP scaffold group compared to the PLGA group, as well as an increase in positive cells with the increase in β-TCP content, indicating that β-TCP significantly enhance osteogenic differentiation. The PLGA/TCP1 group showed an increase (approximately 1.65-fold) ALP activity compared to the PLGA group as well as PLGA/TCP5 (approximately 2.26-fold), while the PLGA/TCP10 group exhibited the highest increase in ALP activity over 14 days (approximately 2.93-fold), as shown in Figure 5b. Formation and deposition of calcium salt nodules regarded as a marker of osteoblast maturation. We examined calcified nodules with alizarin red staining to identify the long-term effects of β-TCP in promoting bone formation [15]. ARS staining resulting from culturing BMSCs after 21 days is shown in Figure 5c. An increased amounts of calcified nodules was observed in the PLGA/TCP10 group, while the PLGA group had the lowest number of calcified nodules. This shows that PLGA/TCP10 extract accelerated and enhanced the mineralization process in osteoblasts, confirming that PLGA/TCP10 promotes osteogenic differentiation of BMSCs.

### 3.6 Effect of PLGA/TCP on angiogenic capacity of HUVECs

**Figure 6:**
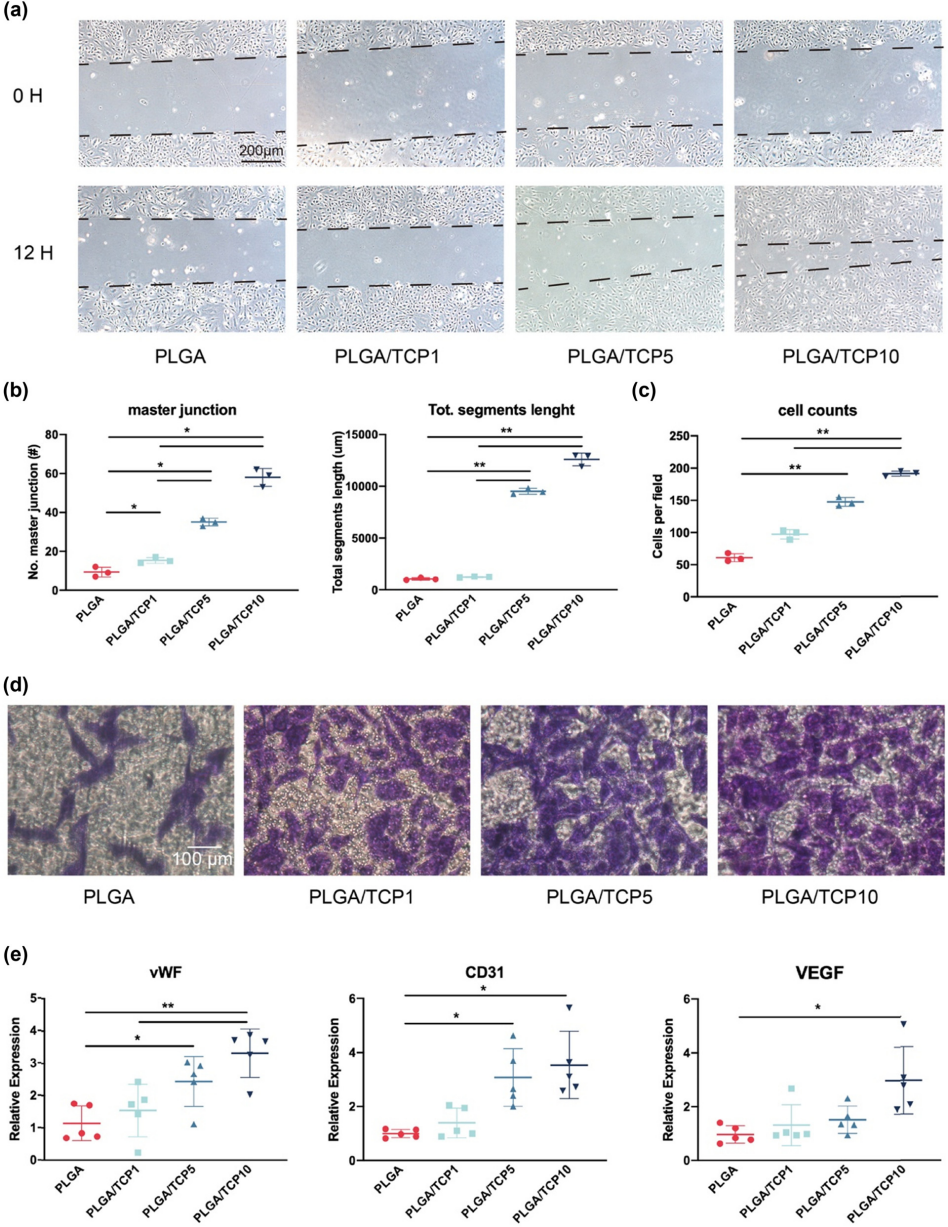
Angiogenic effect of HUVECs treated with extracts. (a) Tube formation assay of HUVECs cells. (b) Number of master junction and total segment length calculated with ImageJ. (c) Quantitative analysis of the Transwell assay. (d) Transwell assay of HUVECs cells. (e) Angiogenesis related gene expression of HUVECs cultured in extracts for 3 days. Data are presented as mean value ± SD; *n* = 3; *p < 0.05 and **p < 0.01.

As shown in Figure 6a, our results displayed that HUVECs in the PLGA/TCP10 group had the best angiogenic capacity. The angiogenic capacity of HUVECs was assessed by ImageJ, by quantifying the number of grids and primary nodes in the tube formation test and thus reflecting the angiogenic capacity of HUVECs, with more primary nodes and total segment length detected in the PLGA/TCP10 group (Figure 6b). Transwell assay was also used to evaluate the effect of PLGA/TCP on the migratory capacity of HUVECs. Results revealed that the crystal violet stain of migrating cells was significantly higher in the PLGA/TCP10 group compared to the other groups (Figure 6c and d). We performed qRT-PCR analysis to validate the angiogenic effect at the gene expression level after 3 days of incubation in the extracts. Compared to cells in the PLGA group, HUVECs in the PLGA/TCP10 group exhibited approximately 3.31-fold vWF expression, 2.96-fold VEGF expression and 3.08-fold CD31 expression (Figure 6e). These results suggest that PLGA/TCP has a positive effect on the angiogenic capacity of HUVECs.

## 4 Discussion

Due to population aging, traffic accident, or sports injury, bone defects and comminuted fracture remain as common clinical challenges [23,24]. The clinical gold standard for the reconstruction of bone defects is autologous bone grafting. However, its application is restricted by the low availability of bone grafts and donor-site morbidity [1,15,23]. Therefore, there is an increasing clinical demand for biomaterials for the treatment of bone defects. β-TCP is favored by researchers because of its excellent biocompatibility, good osteogenic activity, and can be combined with other materials to improve its biomechanical properties and osteogenic capacity [25]. Although the mechanical strength of β-TCP is slightly lower, its biodegradation rate is faster, which facilitates the growth of new bone around the implanted β-TCP base scaffold, and thus, β-TCP can be combined with other materials to improve its biomechanical properties and osteogenic ability [26]. However, the biomedical application of β-TCP is limited due to its inherent brittleness, non-degradability, and non-plastic ability [25,27]. PLGA, authorized by the US FDA, is frequently used to regulate the degradation rate of implants to meet clinical needs [28]. It was reported that the strength of calcium phosphate cement could be enhanced by adding PLGA medical sutures to form connected pores after polymer fiber degradation [29]. Studies also revealed that PLGA nanofibers can greatly improve the toughness and accelerate the degradation of calcium phosphate by using high aspect ratio and controllable degradation of PLGA [15]. PLGA degradation produces GA and LA, which could be metabolized and absorbed by the human body [15]. Previous studies have showed that the acidic products due to PLGA degradation lower the pH of the local environment, producing a local inflammatory response [30]. Simultaneously, the release of β-TCP is able to neutralize the local production of acid to some extent, overcoming the potential negative effects caused by excessive acidity and regulating the microenvironment for osteogenic repair [31]. Moreover, a high level of LA can make the local microenvironment become weakly acidic, which can facilitate the degradation of β-TCP [32,33]. In this study, we found that the viscosity of the bioactive membrane will be too large and the particles are easy to agglomerate when the TCP concentration exceeds 10%. It is difficult to prepare the electrospinning so that we prepared bioactive membrane with three concentrations of PLGA/TCP1, PLGA/TCP5, and PLGA/TCP10 for subsequent experiments in this study. Our results also showed that Ca^2+^ release was greater in the PLGA/TCP10 group than in the other groups, which led to an osteogenic environment during material degradation, thus greatly improving osseointegration and the growth of new bone towards the defect area.

As good biocompatibility is the prerequisite for the application of biomaterials in tissue repair [1], in this study, CCK-8 and live/dead staining assay were applied to evaluated cell viability. In addition, after cells were co-cultured with the surface of the material, it was still observed that both BMSCs and HUVECs could proliferate well and cell viability was not inhibited. Cell survival rate of live/dead assay and CCK-8 analysis showed that BMSCs proliferated well in all groups, demonstrating that PLGA/TCP membranes were non-cytotoxic for cell viability and proliferation. For osteogenic capacity, ALP staining and ALP activity assay were performed to measure the osteogenic capacity of BMSCs after osteogenic induction. ALP is a primary marker reflecting osteoblast differentiation and early bone mineral formation on biomaterials *in vitro* [34]. The results of ALP activity on the days 7 and 14 showed that osteogenic differentiation capacity of BMSCs was significantly increased after adding various membranes’ extracts, and PLGA/TCP10 group had the best osteogenic differentiation capacity in BMSCs. Moreover, the results of ALP staining on the day 14 and ARS staining on the day 21 also showed that the mineralization deposition gradually increased and PLGA/TCP10 group had the most mineralized nodules, suggesting that PLGA/TCP10 had the best effect of osteogenic differentiation capacity on BMSCs.

Bone tissue is a vascularized connective tissue, which primarily deliver oxygen, nutrients, hormones, neurotransmitters, and growth factors to bone cells and such neovascularization is indispensable for proper bone regeneration and remodeling [35–37]. Furthermore, it is reported that LA promotes vascular growth, which is also important for bone regeneration [38]. A recent study has revealed that the vascularization of bone defects is conducive to the recruitment of more BMSCs. Hence, the establishment of a vascular network at the bone defect area is favorable for bone formation [39]. Currently, the influence of PLGA/TCP on the angiogenic capability of HUVECs was also investigated by tube formation tests. The degradation of PLGA releases lactic acid, leading to localized accumulation of lactic acid in the osteogenic region. Moderate levels of lactic acid promote the angiogenic effect of HUVECs, but an overly acidic microenvironment impedes angiogenesis [40]. Regulation of the microenvironmental pH by β-TCP allows for a relatively neutral osteogenic zone, promoting angiogenesis *in vitro*. In the present study, the invasion and angiogenesis ability of HUVECs were assessed by tube formation and Transwell assay. As shown in the results, PLGA/TCP10 membrane had the best angiogenic capacity effect on HUVECs. The number of master junctions and total segment length were used to quantify the tube formation capacity by Image J, and the results also confirmed this tendency. Similar trends were also observed in the transwell assay, where the PLGA/TCP10 had the best invasive capacity of HUVECs. qRT-PCR results also revealed that the PLGA/TCP10 group had the best positive effect on angiogenic capacity in HUVECs. As angiogenesis is closely associated with osteogenesis, taken together, the results of this study demonstrated that PLGA/TCP10 membrane could improve the angiogenesis ability of HUVECs and synergistically promote bone regeneration.

## 5 Conclusion

In this study, we developed a PLGA/β-TCP compound membrane through electrostatic spinning aiming to control the release of β-TCP, to modulate the activity of osteoblasts and vascular endothelial cells. This biodegradable bioactive repair membrane possesses good mechanical and thermochemical properties, offering certain biological functions and is capable of facilitating osteogenesis and angiogenesis *in vitro*. Our results show that PLGA/β-TCP composite membranes further support the potential of PLGA and β-TCP as biomaterials.

## Supplementary Material

Supplementary Figure
