# Supplementary material for: Absorbable calcium and phosphorus bioactive membranes promote bone marrow mesenchymal stem cells osteogenic differentiation for bone regeneration
Source: Open Life Sci. 2024 Apr 16;19(1):20220854. doi: 10.1515/biol-2022-0854 (PMC11022123; doi:10.1515/biol-2022-0854)
Supplement: Supplementary Figure [file biol-2022-0854-sm.pdf]

Supplementary material

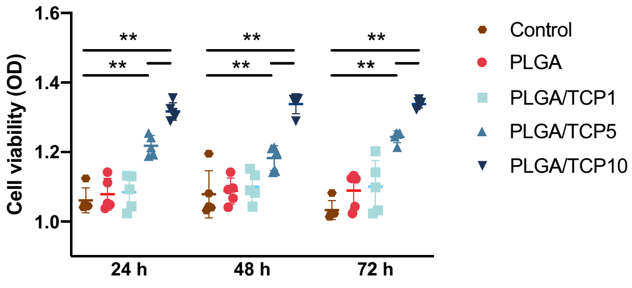

Figure S1: Proliferation of BMSCs treated with extracts at 24, 48 and 72 h, respectively. Data are presented as mean ± SD; $n = 3$; $*p < 0.05$ and $**p < 0.01$.

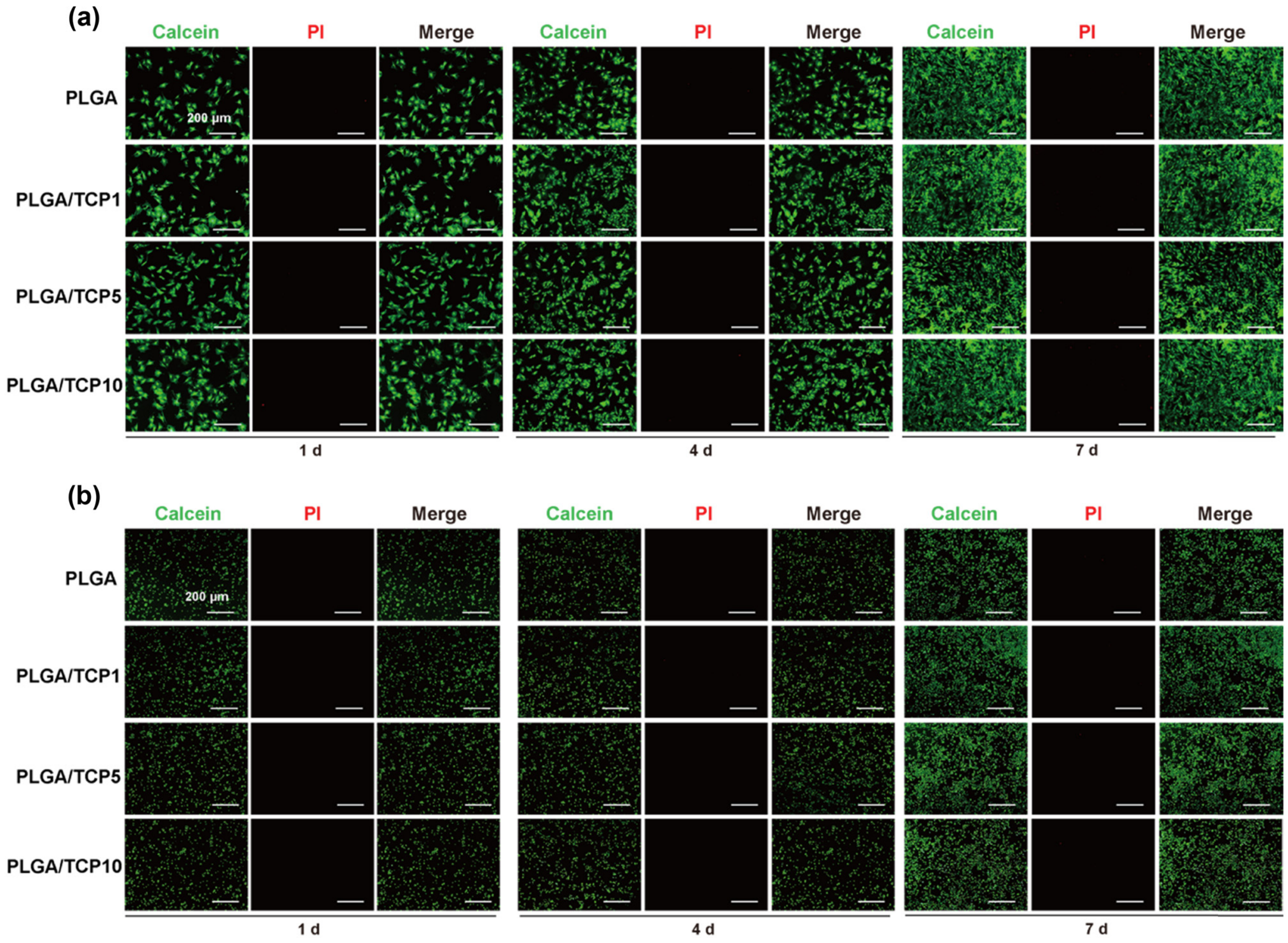

Figure S2: Proliferation and viability of cells co-cultured on the surface of bioactive membranes. (a) Live/Dead staining results of BMSCs; (b) Live/Dead staining results of HUVECs.

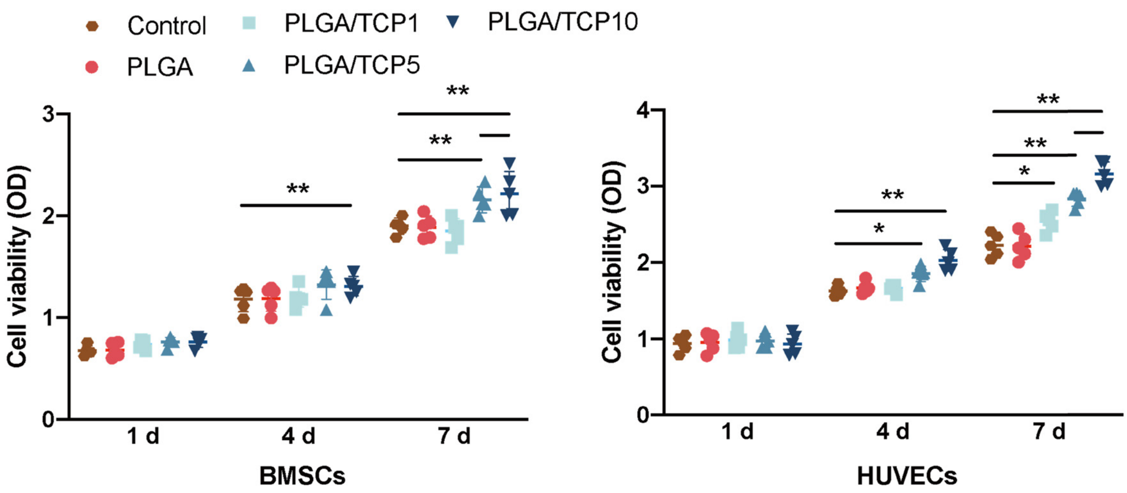

Figure S3: Viability of BMSCs and HUVECs co-cultured on the surface of bioactive membranes. Data are presented as mean ± SD; $n = 3$; *$p < 0.05$ and **$p < 0.01$.
